# CHANGES AMONG COLLEGE STUDENTS’ ATTITUDES, KNOWLEDGE, AND INTENTION TO WORK WITH OLDER ADULTS

**DOI:** 10.1093/geroni/igad104.2697

**Published:** 2023-12-21

**Authors:** Christopher O’Brien, Jessica Strong

**Affiliations:** Providence College, Providence, Rhode Island, United States; University of Prince Edward Island, Charlottetown, Prince Edward Island, Canada

## Abstract

Curricular intervention studies have examined if instruction in aging and gerontology affects undergraduates’ attitudes, knowledge, and perceptions toward older adults. However, less is known about the curricular impact on undergraduates’ intentions to work with older adults and attitudes and knowledge about sexuality and aging. The current longitudinal study examined the impact of an upper-level adult development psychology course on student attitudes, knowledge, and intentions toward working with older adults. Participants were 8 undergraduate students enrolled in upper-level undergraduate psychology courses. Participants completed validated, self-report questionnaires at the beginning of the semester and at the end of the semester related to their attitudes towards working with older adults, ageism attitudes, aging sexual knowledge and attitudes, and attitudes and knowledge about aging. Paired samples t-tests were used to examine changes in main outcome variables. Students endorsed significantly greater attitudes toward working with older adults at the end of the semester (t = -2.57, p = .037). Additionally, knowledge about sexual aging significantly increased (t = 2.62, p = 0.34). These findings suggest curricular interventions may positively impact attitudes toward working with older adults and sexual aging knowledge. Future work will evaluate predictors of change in attitudes, knowledge, and intentions. By identifying factors that increase undergraduates’ intentions to work with older adults and improve attitudes and knowledge regarding sexual aging, we may elucidate meaningful points of intervention to enhance the pursuit of careers in the geriatric workforce.

